# Spontaneous Bile Leak in a Patient Without Recent Abdominal Surgery or Trauma

**DOI:** 10.7759/cureus.16702

**Published:** 2021-07-28

**Authors:** Daniel Micheli, Keshav R Patel, Tong Li, Mahmoud Kassir, Wesley Eichorn

**Affiliations:** 1 Family and Community Medicine, Western Michigan University Homer Stryker School of Medicine, Kalamazoo, USA; 2 Internal Medicine, Western Michigan University Homer Stryker School of Medicine, Kalamazoo, USA

**Keywords:** spontaneous bile leak, biliary tree, bile duct injury, abdominal pain, ventral hernia

## Abstract

Bile leaks are a rare occurrence most often seen as a complication of cholecystectomy. Other less common etiologies include endoscopic retrograde cholangiopancreatography (ERCP), percutaneous transhepatic cholangiography (PTC), liver surgery, percutaneous drainage of liver abscesses, living donor hepatectomy, and non-iatrogenic abdominal trauma. In this case study, we present a 67-year-old female with morbid obesity who presented with abdominal pain and was diagnosed with a spontaneous bile leak. She had no history of recent surgery or abdominal trauma. CT revealed that the patient’s gallbladder was located in the right lower quadrant, most likely due to mass effect from a large ventral hernia, and possible fluid collection extending from the gallbladder along the surface of the anterior inferior right hepatic lobe. Hepatobiliary iminodiacetic acid (HIDA) was performed due to a concern for cholecystitis. HIDA demonstrated a bile leak in the right upper abdomen of unknown etiology. Initially, there was a concern for gallbladder obstruction. Gastroenterology recommended magnetic resonance cholangiopancreatography (MRCP), however, MRCP was not possible due to the patient’s body habitus. The patient had normal liver function tests, was tolerating oral intake, and her abdominal pain resolved, therefore, we became less suspicious of gallbladder obstruction. This case suggests that bile leak should be included in the differential diagnosis for abdominal pain even in patients who have not had recent abdominal surgery or procedures. This case also highlights the unique anatomical finding of a right lower quadrant gallbladder secondary to mass effect from a large ventral hernia.

## Introduction

Bile leaks are a relatively rare occurrence that can result in significant morbidity and mortality. Most bile leaks occur as a complication of cholecystectomy [1]. Only 0.5% of patients that undergo a cholecystectomy develop a bile leak; however, patients with a bile leak are 1.7 times more likely to die within the first post-surgery year [2]. Possible complications of a bile leak include biliary fistula, peritonitis, and subhepatic/subphrenic collections of bile [3]. There is little evidence that bile duct leaks are directly caused by gallbladder disease, including cholecystitis and choledocholithiasis. Other etiologies of bile leaks include endoscopic retrograde cholangiopancreatography (ERCP), percutaneous transhepatic cholangiography (PTC), liver surgeries such as hydatid cyst removal and tumor resection, percutaneous drainage of liver abscesses, living donor hepatectomy, and non-iatrogenic abdominal trauma [4-5]. In this case report, we present a rare case of spontaneous bile leak in a patient with no history of recent surgery or abdominal trauma.

## Case presentation

A 67-year-old female with a past medical history of morbid obesity [body mass index (BMI) of 58.4 with a height of 1.6 m and weight of 149.5 kg] presented with right-sided abdominal pain. The abdominal pain had been present and consistent for several weeks but had increased acutely over the last few hours. She denied fevers, chills, chest pain, palpitations, shortness of breath, cough, nausea, and vomiting. Her past medical history was significant for multiple cardiopulmonary comorbidities including heart failure with preserved ejection fraction, chronic obstructive pulmonary disease requiring 3 L of oxygen per minute at baseline, myocardial infarction, and type 2 diabetes mellitus. Her surgical history was significant for a remote history of a hernia repair, but the patient was unclear of when it occurred.

At the emergency department (ED), her respiratory rate was 32 breaths/minute with an oxygen saturation of 88% on 3 L of oxygen. The rest of her vitals were unremarkable. There were no significant abnormalities on the cardiac exam. She had decreased breath sounds in the lower lung lobes bilaterally with mild diffuse wheezing throughout all lung fields. An abdominal exam revealed moderate tenderness to palpation in the right upper and lower quadrants. A hernia was palpated in the right lower abdomen.

Pertinent lab values included a white blood cell count of 12,400 cells per microliter with negative blood and urine cultures. Basic metabolic panel and lactic acid were unremarkable. Liver tests were within normal limits (Table 1). Chest X-ray was inconclusive because of poor penetration due to the patient’s body habitus.

**Table 1:** Initial liver tests. AST, aspartate aminotransferase; ALT, alanine aminotransferase; ALP, alkaline phosphatase

Lab test	Lab value	Reference range
Albumin	3.6 g/dL	3.5–5.0 g/dL
AST	10 IU/L	0–37 IU/L
ALT	13 IU/L	6–37 IU/L
ALP	111 IU/L	29–117 IU/L
Total bilirubin	0.5 mg/dL	0.0–1.2 mg/dL

**Figure 1:**
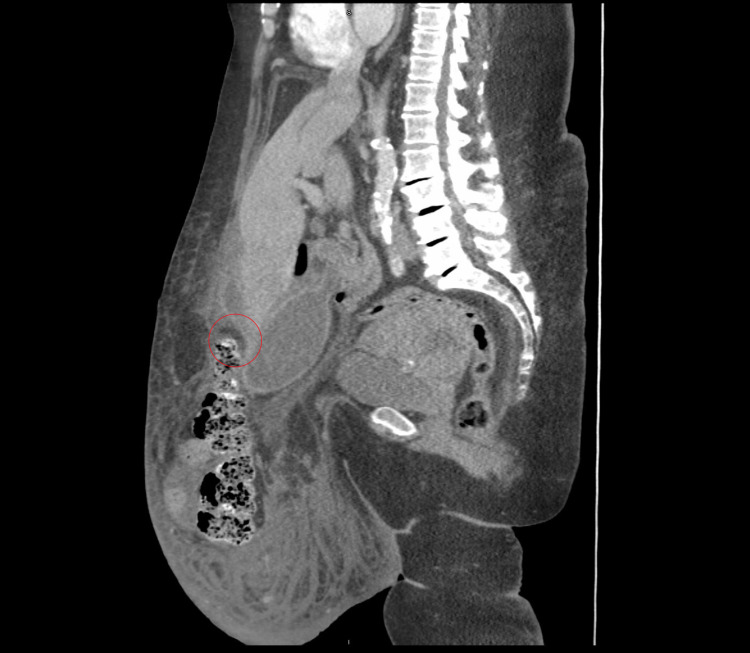
Sagittal CT of the abdomen and pelvis. Sagittal reconstruction of CT abdomen and pelvis done on hospital day 1 demonstrates rectus diastasis with a large hernia consistent of omentum, large and small intestine, right hepatic lobe of the liver, gallbladder, and urinary bladder (not shown). There is ongoing gallbladder distension and thickening. Pericholecystic loculated rim enhancing fluid (red circle) is seen on the anterior inferior right hepatic lobe.

**Figure 2:**
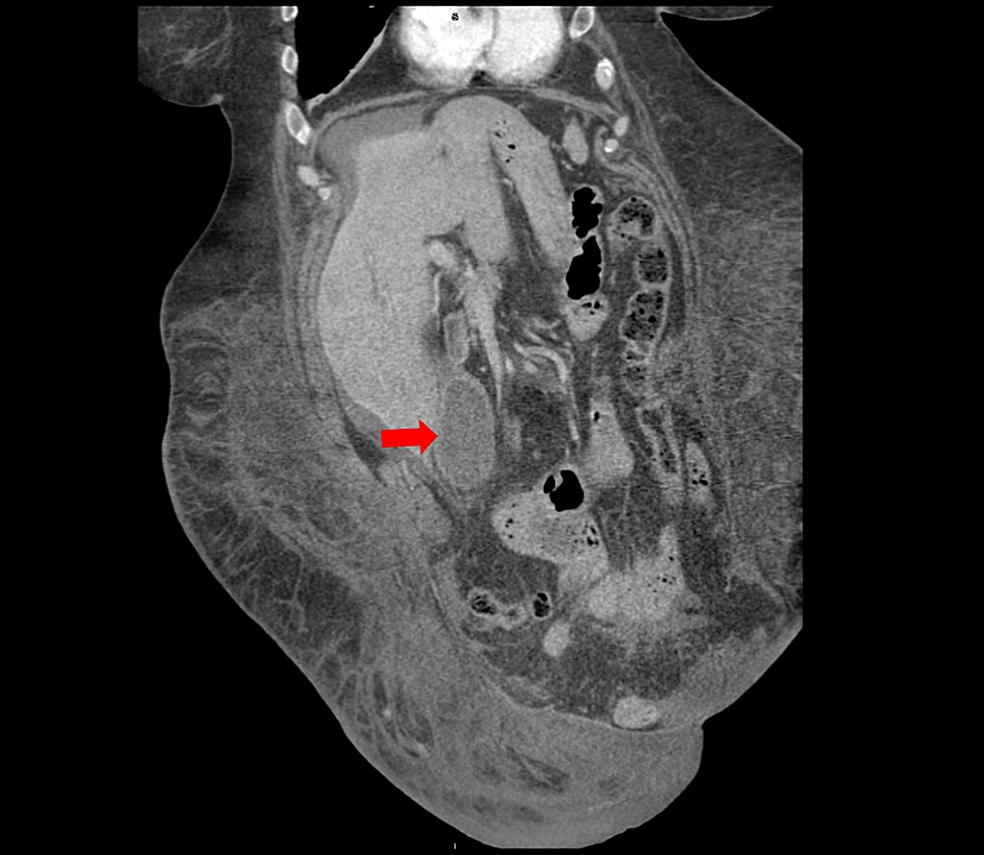
Coronal CT demonstrating the inferior location of the gallbladder. This figure demonstrates the anatomical position of the patient in the supine position. Note the inferior location of the gallbladder (red arrow) is noted to be in the right lower quadrant.

CT of the abdomen and pelvis with contrast showed distension and thickening of the gallbladder wall, pericholecystic stranding, and a fluid collection extending from the gallbladder along the surface of the anterior inferior right hepatic lobe (Figure 1). The gallbladder was located in the right lower quadrant of the abdomen due to the mass effect from a large ventral hernia (Figure 2). No bile duct abnormalities were noted in the radiology report for the CT scan. The leading diagnosis on the differential at the time was acute cholecystitis. The patient received ceftriaxone and metronidazole in the ED and was admitted to the inpatient service.

**Figure 3:**
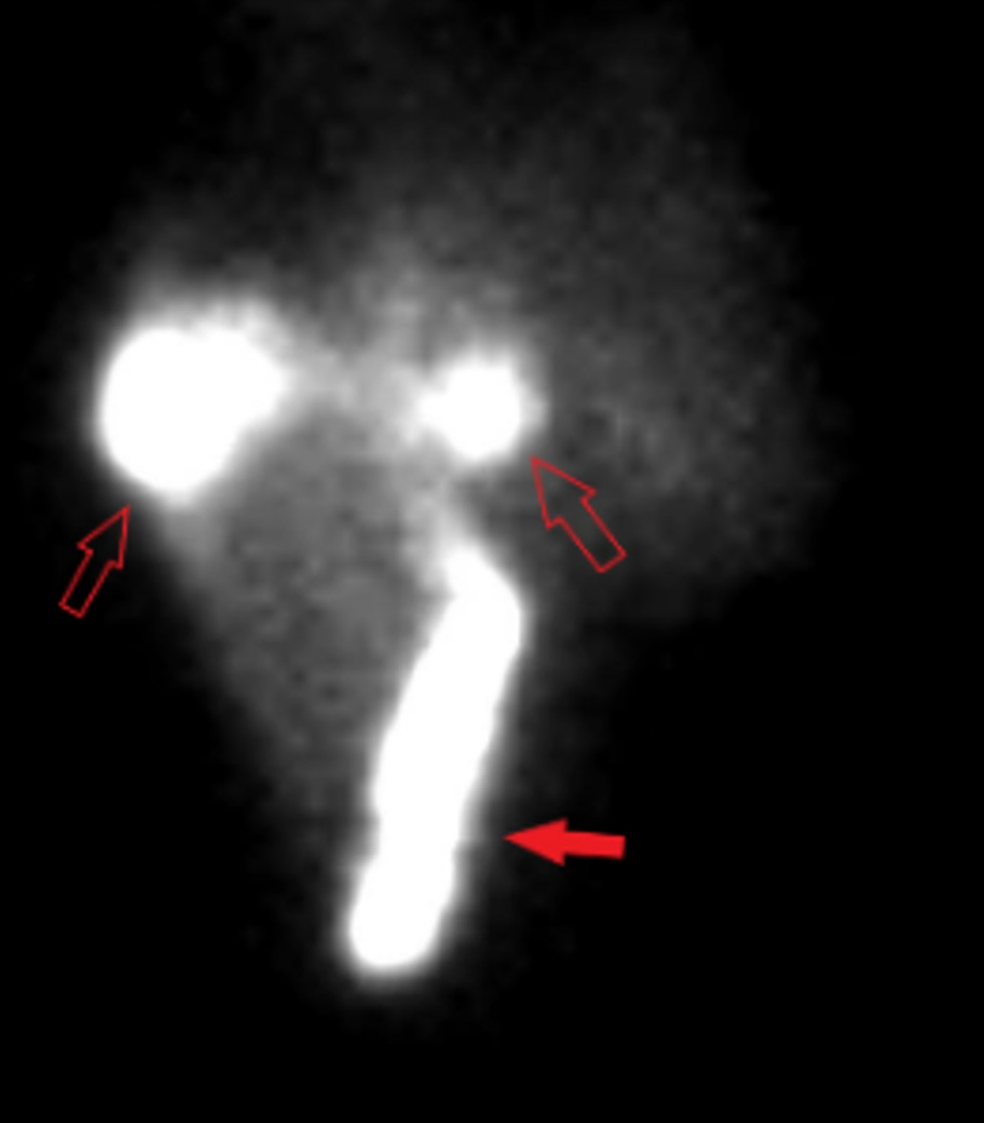
HIDA scan. HIDA performed on hospital day 2 demonstrated abnormal tracer activity (hollow arrows) shown in the right upper abdomen suggestive of a biliary leak. The gallbladder (solid arrow) is located more inferiorly due to ventral wall hernia. HIDA, hepatobiliary iminodiacetic acid

Hepatobiliary iminodiacetic acid (HIDA) scan demonstrated a bile leak in the right upper abdomen of unknown etiology (Figure 3). The patient was not a candidate for surgery or ERCP due to her medical comorbidities. The patient remained afebrile and thus antibiotics were discontinued.

Interventional radiology was consulted for draining the fluid surrounding the gallbladder, and there was a concern for possible common bile duct (CBD) obstruction due to the patient’s abdominal pain, despite no evidence on a CT scan. In addition, interventional radiology stated that since the patient was not a surgical candidate and there were no plans for cholecystectomy in the future, then placement of cholecystostomy tube/drain would likely be lifelong. Gastroenterology recommended magnetic resonance cholangiopancreatography (MRCP) to better delineate the anatomy of the bile ducts and to gauge the odds of successful ERCP. However, MRCP was not possible and there was no MRI within a 100-mile radius that could accommodate our patient’s body habitus.

Given our patient’s continued normal liver function testing and improvement of abdominal pain, we became less suspicious for a gallbladder obstruction. Her abdominal pain resolved by day 8 of the hospital stay and she tolerated oral intake. Management during her hospital stay included bilevel positive airway pressure (BiPAP) for hypercarbic respiratory failure along with bumetanide and metolazone for pulmonary edema. The patient was ultimately discharged to a skilled nursing facility.

## Discussion

Bile leaks have a wide range of morbidity and mortality based on the anatomical location and the size of the leak. Some 70% of bile leaks occur from either minor bile duct injuries or injuries to the accessory bile duct, duct of Luschka, or gallbladder bed and these leaks usually resolve spontaneously with supportive treatment [6]. Some 30% of bile leaks involve major bile duct injury, which is more serious and almost always requires prompt intervention and repair [6].

The typical treatment for a bile duct leak is ERCP with the placement of a temporary bile duct stent. This procedure is minimally invasive and effective in preventing further leakage and reoccurrence. In addition, antibiotics can be given to help prevent abdominal cavity infections. If the leak is severe or does not respond to ERCP, surgery may be needed. Some surgical procedures used to repair bile leaks include hepaticojejunostomy, suturing of the cystic duct, and suturing of the hepatic duct [6]. Our patient was not a candidate for ERCP or surgery due to her respiratory status and medical comorbidities.

Symptoms of bile leaks vary based on severity and anatomical location. Common symptoms include abdominal pain, nausea, vomiting, fever, chills, jaundice, and abdominal distension. Our patient presented with moderate right-sided abdominal pain and lacked any other symptoms of a bile leak. She had no history of cholecystectomy, gallbladder disease, abdominal trauma, ERCP, or liver surgery. She had a history of a remote hernia repair, but the patient did not know additional details.

There are some inconsistencies in the literature when it comes to the definition of a bile leak. Some definitions specify that a bile leak occurs following a surgical intervention on or after post-op day 3, and the International Study Group for Liver Surgery (ISGLS) further grades bile leaks depending on how they change clinical management [7-8]. However, a large body of research defines bile leak as a broader term. Using the PubMed search function, a significant amount of literature was found that referred to “spontaneous bile leak,” a term used to refer to a leakage of biliary fluid that did not follow surgical intervention of any kind [4, 9-13]. Spontaneous bile leak has been defined as a bile leak “where a specific cause remains unidentifiable and is usually a diagnosis of exclusion” [9]. One article presented a case similar to ours, of a spontaneous bile leak with no recent surgery, abdominal trauma, or evidence of cholelithiasis or sepsis. They stated that “a bile leak in the absence of recent surgery or a history of abdominal trauma is a rare occurrence,” and used the term “spontaneous bile leak” to describe the incident [12]. Although bile leaks most commonly follow cholecystectomies and other surgical interventions, the definition of the term “bile leak” is not confined to post-surgical complications, as evidenced by a number of articles referring to “spontaneous bile leak” where no recent surgeries took place.

Because this patient underwent hernia surgery in the remote past, it is extremely unlikely that this episode is associated with that surgery. With the lack of any precipitating factors, we believe that this patient’s bile leak was spontaneous. During the hospital workup, CT revealed that the patient’s gallbladder was located in the right lower quadrant (Figure 2). In addition, the patient was morbidly obese with an oversized pannus. The weight from the pannus could be putting an excessive amount of pressure on the gallbladder and liver, which may have increased the risk of a spontaneous bile leak. Our patient’s abdominal pain resolved spontaneously, and she has not returned to the hospital in the last three months.

## Conclusions

We hope to emphasize that bile leaks, although usually seen as a complication of cholecystectomy, have a wide variety of etiologies and can even be seen spontaneously. Major bile duct injuries are serious and must be recognized and treated promptly. Therefore, bile leaks should be included on the differential when patients present with symptoms of abdominal pain, even in the absence of traditional risk factors. Our patient's obesity and large ventral hernia may have increased the risk of a spontaneous bile leak. In addition, we present the unique anatomical finding of a right lower quadrant gallbladder, which could be a risk factor for a spontaneous bile leak.
